# Supplementary material for: Dietary Diversity and Micronutrients Adequacy in Women of Childbearing Age: Results from ELANS Study
Source: Nutrients. 2020 Jul 4;12(7):1994. doi: 10.3390/nu12071994 (PMC7400493; doi:10.3390/nu12071994)
Supplement: Supplementary file 1 [file nutrients-12-01994-s001.pdf]

Table S1. Mean nutrients adequacy ratio of specific nutrients of women of childbearing age by country. ELANS 2014–2015.

	**Argentina**	**Brazil**	**Chile**	**Colombia**	**Costa Rica**	**Ecuador**	**Peru**	**Venezuela**	**ELANS**
	***n* = 521**	***n* = 798**	***n* = 345**	***n* = 464**	***n* = 309**	***n* = 324**	***n* = 480**	***n* = 463**	***n* = 3704**
Vitamin E	0.040 ± 0.01	0.019 ± 0.01	0.024 ± 0.01	0.043 ± 0.02	0.027 ± 0.01	0.051 ± 0.02	0.030 ± 0.01	0.020 ± 0.01	0.031 ± 0.02
Vitamin D	0.307 ± 0.14	0.192 ± 0.11	0.303 ± 0.17	0.457 ± 0.20	0.255 ± 0.13	0.564 ± 0.23	0.521 ± 0.21	0.272 ± 0.12	0.343 ± 0.21
Calcium	0.928 ± 0.25	0.449 ± 0.49	0.553 ± 0.49	0.928 ± 0.25	0.417 ± 0.49	0.904 ± 0.29	0.545 ± 0.49	0.831 ± 0.37	0.684 ± 0.46
Folate	0.715 ± =.19	0.792 ± 0.18	0.649 ± 0.18	0.652 ± 0.15	0.665 ± 0.17	0.730 ± 0.16	0.664 ± 0.16	0.663 ± 0.16	0.702 ± 0.18
Magnesium	0.701 ± 0.15	0.709 ± 0.18	0.648 ± 0.15	0.901 ± 0.14	0.784 ± 0.18	0.909 ± 0.12	0.885 ± 0.13	0.833 ± 0.16	0.788 ± 0.18
Vitamin C	0.860 ± 0.20	0.799 ± 0.24	0.876 ± 0.16	0.950 ± 0.12	0.916 ± 0.16	0.896 ± 0.17	0.955 ± 0.10	0.852 ± 0.18	0.873 ± 0.21
Vitamin A	0.705 ± 0.23	0.834 ± 0.26	0.802 ± 0.23	0.956 ± 0.12	0.843 ± 0.22	0.993 ± 0.05	0.953 ± 0.12	0.955 ± 0.12	0.879 ± 0.19
Piridoxine	0.972 ± 0.07	0.947 ± 0.11	0.955 ± 0.10	0.993 ± 0.06	0.954 ± 0.12	0.996 ± 0.03	0.995 ± 0.03	0.985 ± 0.06	0.973 ± 0.08
Zinc	0.997 ± 0.03	0.961 ± 0.10	0.945 ± 0.10	0.991 ± 0.06	0.957 ± 0.10	0.981 ± 0.05	0.985 ± 0.06	0.982 ± 0.06	0.977 ± 0.07
Iron	0.993 ± 0.03	0.912 ± 0.19	0.984 ± 0.30	0.998 ± 0.02	0.991 ± 0.40	0.999 ± 0.00	0.997 ± 0.52	0.991 ± 0.04	0.977 ± 0.08
Phosphorous	0.983 ± 0.07	0.964 ± 0.10	0.967 ± 0.09	0.988 ± 0.06	0.960 ± 0.11	0.992 ± 0.06	0.985 ± 0.06	0.982 ± 0.07	0.977 ± 0.08
Cobalamin	0.982 ± 0.09	0.973 ± 0.10	0.956 ± 0.12	0.987 ± 0.09	0.988 ± 0.06	0.989 ± 0.05	0.986 ± 0.06	0.985 ± 0.07	0.980 ± 0.09
Cobre	0.998 ± 0.02	0.974 ± 0.08	0.959 ± 0.09	0.993 ± 0.06	0.972 ± 0.08	0.998 ± 0.01	0.998 ± 0.02	0.965 ± 0.09	0.982 ± 0.07
Niacin	0.997 ± 0.02	0.992 ± 0.04	0.994 ± 0.04	1.000 ± 0.00	0.994 ± 0.04	1.000 ± 0.00	0.997 ± 0.04	0.998 ± 0.02	0.997 ± 0.03
Riboflavin	0.998 ± 0.02	0.973 ± 0.08	0.979 ± 0.07	0.995 ± 0.05	0.987 ± 0.05	0.986 ± 0.05	0.993 ± 0.04	0.992 ± 0.04	0.988 ± 0.06
Thiamin	0.998 ± 0.01	0.962 ± 0.09	0.985 ± 0.06	0.996 ± 0.04	0.994 ± 0.03	0.999 ± 0.00	0.999 ± 0.00	0.997 ± 0.03	0.989 ± 0.05
Selenium	0.999 ± 0.00	0.997 ± 0.02	0.998 ± 0.02	0.997 ± 0.05	0.997 ± 0.02	1.000 ± 0.00	1.000 ± 0.00	0.999 ± 0.00	0.998 ± 0.02
MAR	0.834 ± 0.04	0.791 ± 0.08	0.799 ± 0.07	0.872 ± 0.05	0.806 ± 0.07	0.882 ± 0.03	0.852 ± 0.05	0.841 ± 0.04	0.831 ± 0.07

MAR: Mean Adequacy ratio for all 17 micronutrients.
